# Identifying Priority Areas for Conservation and Management in Diverse Tropical Forests

**DOI:** 10.1371/journal.pone.0089084

**Published:** 2014-02-14

**Authors:** Karel Mokany, David A. Westcott, Soumya Prasad, Andrew J. Ford, Daniel J. Metcalfe

**Affiliations:** 1 Ecosystem Sciences, Commonwealth Scientific and Industrial Research Organisation, Canberra, ACT, Australia; 2 Ecosystem Sciences, Commonwealth Scientific and Industrial Research Organisation, Atherton, QLD, Australia; 3 Centre for Ecological Sciences, Indian Institute of Science, Bangalore, India; 4 Ecosystem Sciences, Commonwealth Scientific and Industrial Research Organisation, Dutton Park, QLD, Australia; Cirad, France

## Abstract

The high concentration of the world’s species in tropical forests endows these systems with particular importance for retaining global biodiversity, yet it also presents significant challenges for ecology and conservation science. The vast number of rare and yet to be discovered species restricts the applicability of species-level modelling for tropical forests, while the capacity of community classification approaches to identify priorities for conservation and management is also limited. Here we assessed the degree to which macroecological modelling can overcome shortfalls in our knowledge of biodiversity in tropical forests and help identify priority areas for their conservation and management. We used 527 plant community survey plots in the Australian Wet Tropics to generate models and predictions of species richness, compositional dissimilarity, and community composition for all the 4,313 vascular plant species recorded across the region (>1.3 million communities (grid cells)). We then applied these predictions to identify areas of tropical forest likely to contain the greatest concentration of species, rare species, endemic species and primitive angiosperm families. Synthesising these alternative attributes of diversity into a single index of conservation value, we identified two areas within the Australian wet tropics that should be a high priority for future conservation actions: the Atherton Tablelands and Daintree rainforest. Our findings demonstrate the value of macroecological modelling in identifying priority areas for conservation and management actions within highly diverse systems, such as tropical forests.

## Introduction

Tropical rainforests contain more than 60% of all known species, despite covering only 7% of the earth’s surface [1], [2]. The concentration of diversity in tropical forests endows these ecosystems with particular importance for retaining global biodiversity, yet also presents significant challenges for ecology and conservation science. Understanding patterns in tropical forest diversity and subsequently identifying priorities for conservation is severely hampered by the vast numbers of species present, many of which are rare (or yet to be discovered) and for most of which we have little information regarding their attributes or current distributions [3], [4]. The combination of high diversity and major shortfalls in our knowledge of tropical forest biodiversity limits the applicability of commonly applied species-level modelling approaches [5] in improving our understanding of current patterns in diversity.

As a consequence of the challenges faced in understanding diversity in tropical forests, ecologists have often relied upon community-level approaches to improve our knowledge of these systems. This trend is typified in the Australian Wet Tropics, the largest area of tropical rainforest in Australia [6]. Here, a long history of ecological research has applied and refined structural and compositional classification of tropical plant diversity into forest “types”, “categories”, or “regional ecosystems” [7]–[12]. Across the entire Australian Wet Tropics, these forest categories have been mapped at a fine spatial resolution [12], [13], and subsequently related to underlying environmental variables [14], [15].

In the Australian Wet Tropics, simplifying the significant plant diversity into forest categories has enabled valuable research which has greatly improved our knowledge of these systems. For example, a number of studies have applied correlative modelling to project the distribution of different forest categories back in time, using reconstructed climate conditions, identifying possible rainforest refugia at the last glacial maximum [14], [16]–[18]. Other research has projected the future distribution of forest categories under alternative scenarios of climate change, to identify the likely threat posed by global warming for these tropical forests [19], [20]. Projections for the Australian Wet Tropics under both past and future climates in general show dramatic reductions in the spatial extent of most tropical rainforest categories as precipitation decreases [16].

Despite the insights provided, there are limits in the utility of a community classification approach to understand patterns and identify conservation priorities within diverse tropical forests. A community-level classification approach inherently ignores any variation in diversity within a community category, whilst assuming significant and uniform changes in diversity between categories [21]. Applying community-level categorical data to identify conservation and management priorities could therefore be heavily influenced by the number of categories applied, the relative differences between categories and the basis for the categorisation (e.g. structural v.s. compositional).

In moving beyond community classification approaches, macroecological modelling offers significant potential to both overcome shortfalls in our knowledge of biodiversity in tropical forests, while providing new insight into core patterns of diversity. Specifically, models of species richness (*α*-diversity) and pairwise compositional dissimilarity (*β*-diversity) can provide unique insight, by relating community diversity in a continuous manner to underlying environmental gradients [22], [23]. Models of species richness and compositional dissimilarity already have demonstrated utility in improving our understanding of current diversity patterns within tropical forests [24], [25]. These models of core elements of diversity can also be combined in a new approach (*Dynamic*FOAM), that predicts the composition of every community (i.e. grid cell) over a region, even where available knowledge is limited [26]. Application of these continuous macroecological modelling approaches provides significant potential to improve both our knowledge of the key drivers of tropical forest diversity and our capacity to identify priorities for conservation within these diverse systems.

Here we apply macroecological modelling to identify priority areas for conservation and management of plant diversity within the Australian Wet Tropics. We develop models of species richness and compositional dissimilarity for the 4,313 vascular plant species, then apply the *Dynamic*FOAM algorithm [26] to predict the composition of every community (grid cell) across the region (>1.3 million communities). We analyse these macroecological predictions in a number of novel ways to identify areas of high importance for conservation and management within these diverse tropical forests.

## Materials and Methods

### Spatial Information

**Figure 1:**
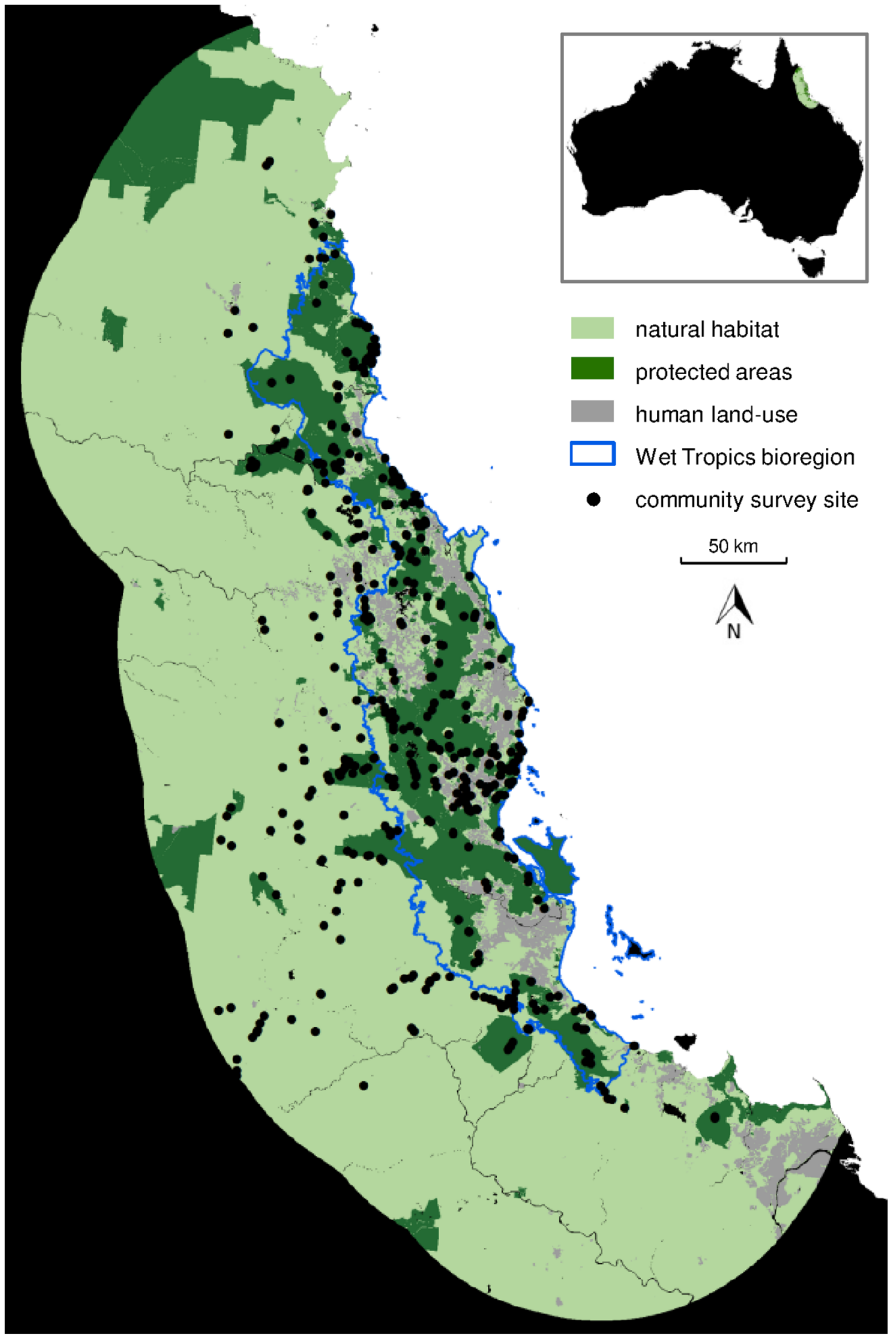
The study region. The study region, being the Wet Tropics Bioregion (blue line) and 100 km of surrounding area, in Queensland, Australia (inset). Protected areas (dark green), unprotected natural habitat (pale green) and modified habitat (grey) are shown, along with the locations of the 527 plant community surveys used in our analyses (black dots).

Our study region (Fig. 1) was defined as the spatial domain including the Wet Tropics Bioregion in Queensland, Australia (from IBRA, 2012), and a 100 km buffer around it (approximately 14°39′S to 20°24′S and 143°54′E to 147°30′E). This region comprises an area of 87,365 km^2^, of which 95.2% is natural vegetation (as classified by Queensland Land Use Mapping, 1999), while the area protected for conservation is 17.6% of the study region and 44.2% of the Wet Tropics Bioregion (as defined by CAPAD, 2010). Our analyses were carried out on a 250 m resolution spatial grid over this region, as defined by the Australian GEODATA 9 Second Digital Elevation Model – V3.

### Biological Data

Plant community survey data over the study region were obtained from two sources: the Queensland Herbarium’s CORVEG survey data [27], and; plant community surveys conducted by CSIRO. The methods applied by the Queensland Herbarium in surveying plant community composition are described in detail in Neldner *et al*. [27], and involved identifying all vascular plant species within a 10×50 m survey plot. In total, 517 Queensland Herbarium survey plots occurred within the study region, however, many of these plots contained species which were only identified to family or genus level, which is inappropriate for accurately quantifying species richness and compositional dissimilarity. We therefore removed those 298 survey plots where more than 10% of taxa that were not identified to the species level. There were no obvious spatial biases in the locations of the survey plots removed (Fig. S1). For the remaining 219 Queensland Herbarium survey plots, those taxa not identified to species level were removed from the analysis. This equated to an average of 1.97 records (range: 1–7) removed across 136 sites containing unidentified taxa. Again, there was no obvious spatial bias in the proportion of records removed from the retained Queensland Herbarium plots, though there was a slight trend for plots with more records to contain a larger proportion of unidentified taxa (Fig. S2).

Plant community surveys conducted by CSIRO between 2004 and 2012 followed a similar methodology to the Queensland Herbarium surveys, but were located in areas that would complement the Queensland Herbarium surveys, and were comprised of 20×50 m plots [28]. Survey data were obtained by CSIRO for 308 sites, and as with the Queensland Herbarium data, we removed those taxa not identified to species level. Almost all taxa in the CSIRO surveys were identified to species level (or to recognised HISPID names where specifies are yet to be formally described), therefore no sites needed to be removed due to coarse taxonomic identification.

Combining community surveys from the Queensland Herbarium and CSIRO gave a total of 527 community survey plots used to generate models of vascular plant species richness and compositional dissimilarity (Fig. 1). We standardised taxonomic nomenclature across these two data sets to be consistent with the Australian Plant Name Index. For both the Queensland Herbarium and CSIRO plant community surveys, we removed all non-native species, as defined by the Census of the Queensland Flora [29] and the Australian Plant Name Index. The analyses we present are therefore relevant to native plant biodiversity only.

For application of the *Dynamic*FOAM procedure (see below), we supplemented the community survey data described above with 104,831 plant species occurrence records, obtained from the Atlas of Living Australia on May 16, 2012. All plant occurrence records obtained were recent (recorded since 1990), spatially accurate (<300 m uncertainty), and consistent with the taxonomic nomenclature applied for the plant community survey data. For the *Dynamic*FOAM procedure, we also included the records of the identified species from the 298 Queensland Herbarium survey plots that were excluded from the community modelling analyses.

### Environmental Data

For the correlative modelling of plant community species richness and compositional dissimilarity, we utilised complete spatial environmental data across the study region. We applied the 250 m digital elevation model of the study region in ANUCLIM [30] to obtain climate data (precipitation, evaporation, temperature, radiation (adjusted for slope & aspect)) and bioclimatic variables (plant growth indices) across the spatial grid (averages for the period 1976 to 2005). We also applied a range of spatial geological (mean geological age, weathering index) and soil (depth, nutrients, bulk density, water holding capacity) data layers, obtained from various sources [31]–[34].

### Scaling Community Diversity from Plot to Grid Cell

It is possible and commonly practised to generate models of species richness and compositional dissimilarity that are relevant to smaller ecological survey plots occurring within larger spatial grid cells. However, for the purposes of the present study, our objective was to generate predictions of species richness and compositional dissimilarity that are relevant to the spatial grain of the 250 m grid cells (62,500 m^2^) rather than the much smaller area that the survey plots occupy (500–1,000 m^2^). To transform our community diversity data so that they were relevant to the spatial grid cell resolution, we applied a recently developed biodiversity scaling method [35].

Briefly, this approach applies the species-area power model (*S* = *cA^z^*) to scale both species richness and compositional dissimilarity from small sample areas to larger areas. Under this approach, the species richness of a grid cell (*S*) is predicted from the observed richness of the community survey (*c*) and the area of the grid cell relative to the survey area (e.g. *A* = 62,500 m^2^/500 m^2^ for the Queensland herbarium data). To scale pair-wise compositional dissimilarity from the community surveys to the grid cells they occurred within, we first predict the number of species in common between the two grid cells *i* and *j* (*S_com,ij_*) from the observed number of species in common between the two community surveys (*c_com,ij_*) using the species area power relationship (*S_com,ij_* = *c_com,ij_A^zcom^*). We then calculate the predicted Sorensen’s compositional dissimilarity between the two grid cells (*β_ij_* = 1– [2*S_com,ij_*/(*S_i_*+*S_j_*)]) using the predicted species richness of each grid cell (*S_i_*, *S_j_*) and the predicted number of species in common between the two grid cells (*S_com,ij_*). We applied a single scaling factor for species richness (*z* = 0.25) and for the number of species shared between a pair of communities (*z_com_* = 0.42), as derived previously [35]. This scaling approach retains the underlying gradients in species richness and compositional dissimilarity that are observed across the community survey plots, but scales the absolute values so that they better represent those of the grid cells being modelled.

### Species Richness Modelling

We generated a model of plant species richness using the Generalized Regression and Spatial Prediction package (GRASP [36]) in R [37]. GRASP applies a generalised additive modelling framework, and for the current implementation we used a Poisson link function with 3 degrees of freedom for each independent variable. We applied an interactive backward variable selection process, using a range of environmental variables that we hypothesised could be important in influencing plant community species richness in the study region. Candidate environmental variables were gradually omitted based on model Bayesian Information Criterion values, variable significance, and variable contribution to deviance reduction. Plant community species richness for each cell on the spatial grid of the region was predicted using the final model and the environmental variables for every grid cell.

### Compositional Dissimilarity Modelling

We generated a model of pair-wise plant community compositional dissimilarity (Sorensen’s dissimilarity) using Generalised Dissimilarity Modelling (GDM) which is an extension of matrix regression, designed to accommodate both the curvilinear relationship of observed compositional dissimilarity with increasing ecological distance between sites, and the variation in the rate of compositional turnover at different positions along environmental gradients [23]. We applied an interactive backward variable selection process, using a range of environmental variables that we hypothesised could be important in influencing plant community compositional dissimilarity in the study region. We applied custom written code in R [37] to implement a permutation test of variable and model significance. Using this procedure with 1,000 permutations, candidate environmental variables were gradually omitted based on variable significance and contribution to deviance reduction. We used the final model of compositional dissimilarity to generate spatially complete transformed environmental layers, which allow prediction of compositional dissimilarity between any pair of communities (i.e. pairs of cells on the spatial grid) [38].

### Predicting Community Composition with *Dynamic*FOAM

We applied the *Dynamic*FOAM procedure to generate predictions of the composition of each community (i.e. grid cell) across the region. *Dynamic*FOAM is an optimisation algorithm that constructs species lists for each community which best meet the constraints of modelled estimates of the number of species present, the predicted dissimilarity in species composition between each pair of communities and any available data on the occurrences of specific species at specific sites [26]. When predicting the composition of all communities using *Dynamic*FOAM, it is possible to include a specified number of hypothetical species (e.g. undescribed species) [26], however, for the current implementation we applied only the 4,313 native species whose occurrences had been recorded within the study region. Given the stochastic nature of the *Dynamic*FOAM algorithm, we generated 10 replicate solutions, with the results presented here being summaries over these replicates.

### Analysing Predictions of Community Composition

We applied a number of analytical approaches in examining our predictions of community composition across the study region. The median area of occurrence of species within each community (grid cell) was quantified by combining the predicted composition of each community and the predicted area of occurrence of each species over the study region. We also determined the predicted number of species within each community that were endemic to a circular area of radius 30 km centred on that community. We calculated the predicted number of “primitive” angiosperm families represented in each community by combining predicted community composition with family-level taxonomic affinity. Primitive angiosperm families were defined as phylogenetically near-basal, and included the families Austrobaileyaceae, Myristicaceae, Himantandraceae, Eupomatiaceae, Annonaceae, Atherospermataceae, Calycanthaceae, Hernandiaceae, Lauraceae, Monimiaceae, Winteraceae, Aristolochiaceae and Piperaceae [6], [39]. Each of the above indices provide alternative measures of the conservation value of each community (grid cell), and many additional measures of conservation value could also have been obtained from our analyses. Here we simply demonstrate how these four alternative measures of conservation value can be synthesised into a single index of conservation value, by first normalising each to a 0–1 range (where 1 = highest conservation value), then calculating the mean value across all four normalised conservation attributes for each community.

## Results

**Figure 2:**
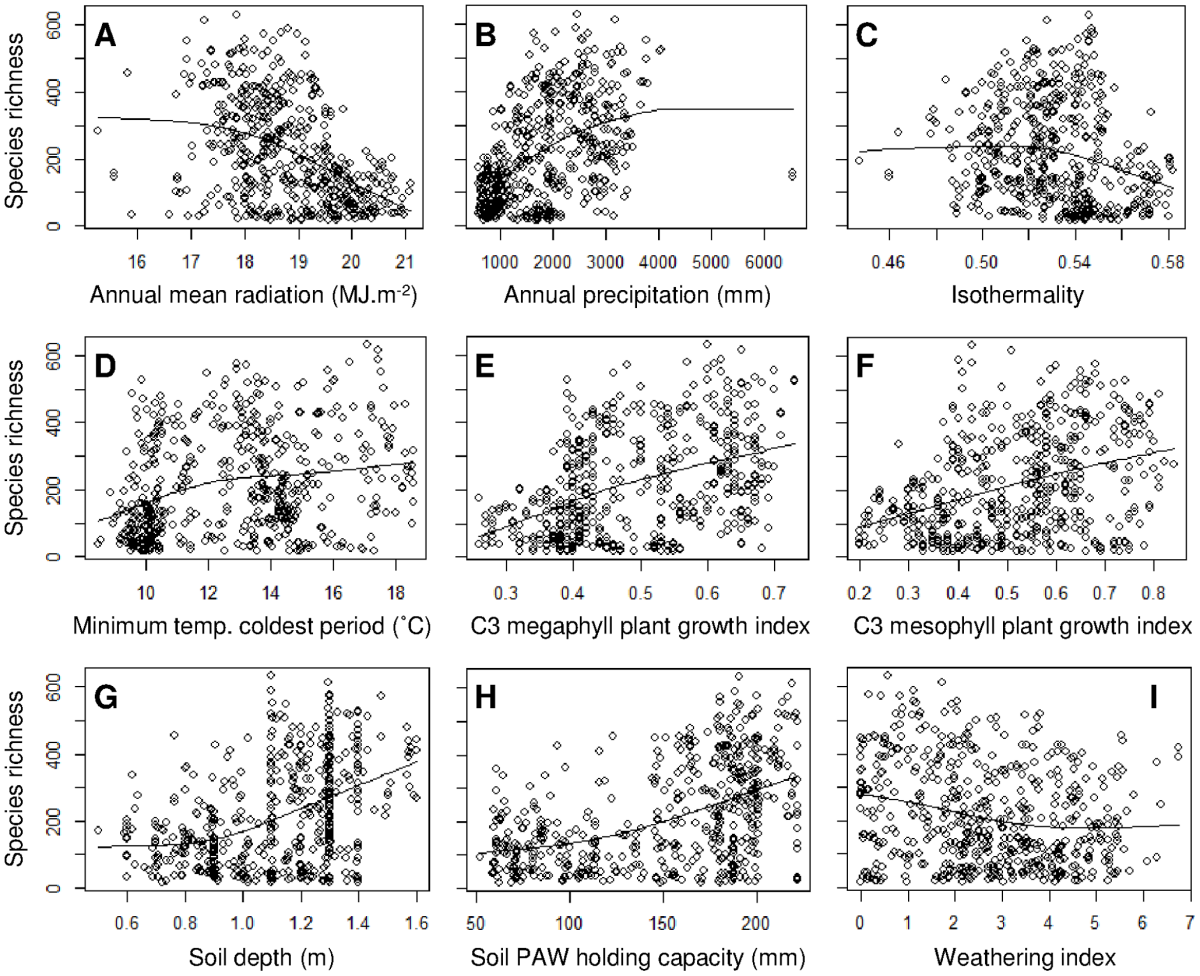
Species richness model response functions. Plant species richness as a function of each of the nine explanatory variables included in the model of plant species richness ((*A*)–(*I*)). The line shows the fitted relationship in the model, while observed data for the 527 community survey sites are shown by the open circles (richness scaled to grid cell area).

**Figure 3:**
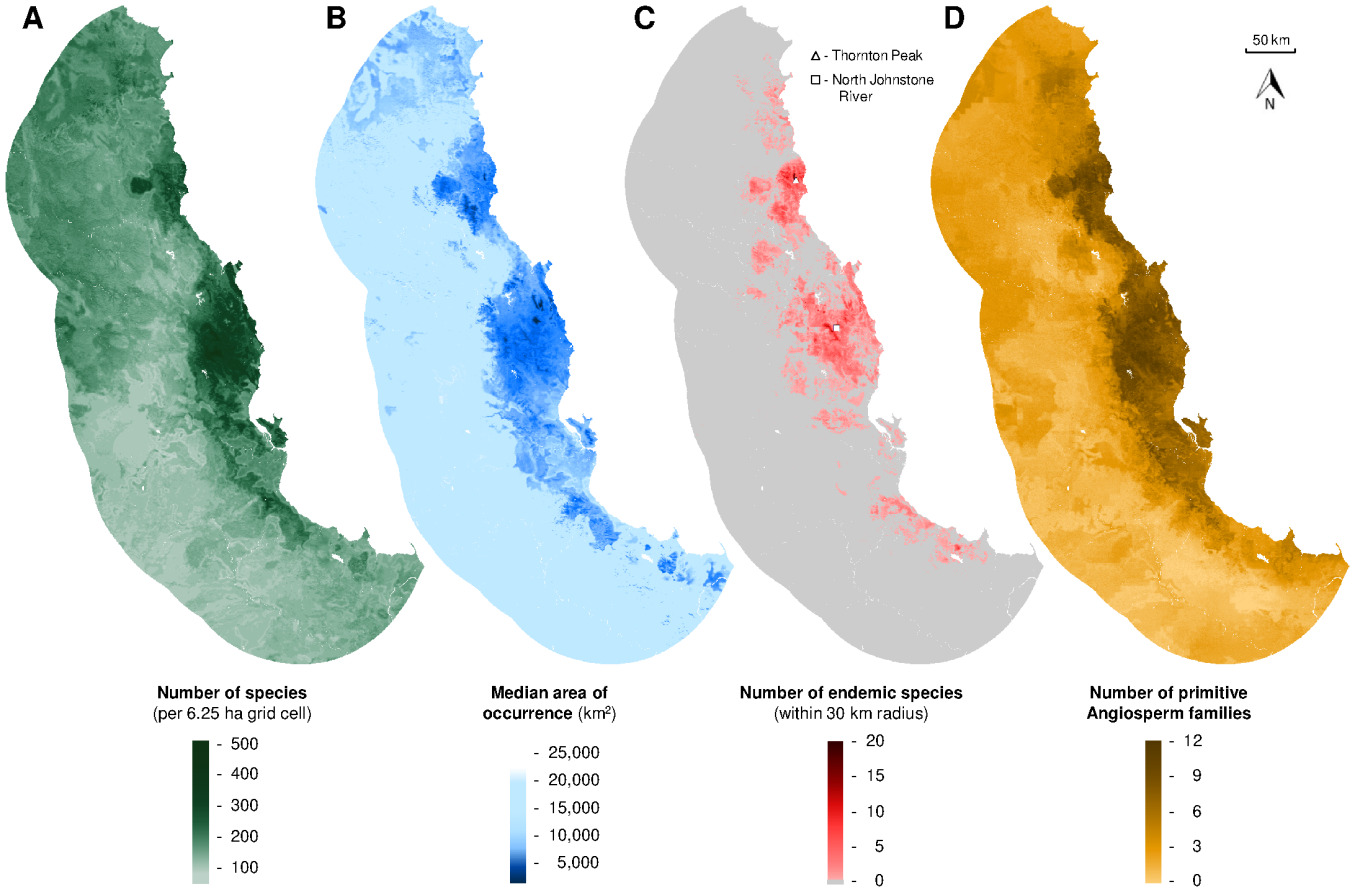
Predicted plant diversity attributes. Predictions across the study region of: (*A*) species richness; (*B*) median area of occurrence of species within each community; (*C*) the number of species present that are endemic to a circular area of radius 30 km centred on each community, and; (*D*) the number of primitive angiosperm families represented in each community (250 m grid cell).

The model of plant species richness in the Australian Wet Tropics explained 40.7% of the total deviance (D^2^), and included nine independent variables of climate and substrate (Table 1, Fig. 2). The strongest relationships with individual environmental variables were for species richness to increase with annual precipitation (D^2^ = 25.1%; *P*<0.001), soil plant available water holding capacity (D^2^ = 22.5%; *P*<0.001), C3 megaphyll plant growth index (D^2^ = 19.5%; *P*<0.001) and decrease with annual mean radiation (D^2^ = 21.9%; *P*<0.001) (Table 1, Fig. 2). Spatial projection of the species richness model showed moist coastal mountainous areas as having the greatest predicted species richness, while communities where predicted richness was lowest occurred in the drier inland areas in the south of the region (Fig. 3A).

**Table 1:** Variable contribution to the Generalised Additive Model of plant species richness for the Australian Wet Tropics.

	All variable model	Single variable model
Variable	% deviance lost	% deviance explained
Annual mean radiation	0.8**	21.9**
Annual precipitation	0.4**	25.1**
Isothermality	0.4**	5.0**
Minimum temp. coldest period	2.5**	11.7**
C3 megaphyll growth index	3.4**	19.5**
C3 mesophyll growth index	3.4**	12.7**
Soil depth	5.0**	17.9**
Soil PAW holding capacity	9.0**	22.5**
Weathering index	6.1**	6.8**

**P<0.001; PAW = Plant available water; temp. = temperature.

For the full model of plant species richness (all variable model), the null deviance = 44,424, the residual deviance = 26,359, the residual degrees of freedom = 499, the deviance explained = 40.7% and the model *P*-value <0.001.

**Figure 4:**
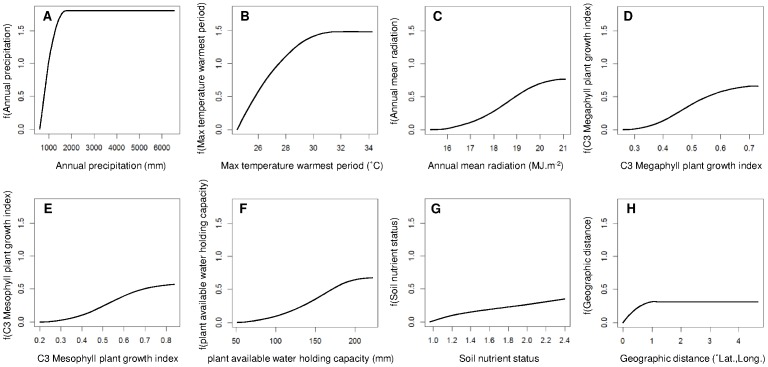
Compositional dissimilarity model response functions. GDM transformed environmental values as a function of the original environmental values for each of the eight explanatory variables included in the model of community compositional dissimilarity ((*A*) – (*H*)). The relative y-axis (transformed) range for the response of each variable indicates the relative strength of that variable in determining compositional dissimilarity, while the nonlinearity of the response indicates which sections of the environmental gradient have steeper predicted compositional dissimilarity.

The model of compositional dissimilarity for plant communities in the Australian Wet Tropics included seven independent climate and substrate variables, plus geographic distance, and explained 34.2% of the total deviance (Table 2, Fig. 4). The strongest relationships between compositional dissimilarity and individual environmental variables were for annual precipitation (D^2^ = 23.8%; *P*<0.001), annual mean radiation (D^2^ = 16.6%; *P*<0.001) and C3 megaphyll plant growth index (D^2^ = 16.7%; *P*<0.001) (Table 2, Fig. 4). In the full model with all eight variables, the amount of deviance in compositional dissimilarity explained solely by geographic distance was relatively low (lost D^2^ when removed = 0.3%; Table 2, Fig. 4H).

**Table 2:** Variable contribution to the Generalised Dissimilarity Model of pairwise community compositional dissimilarity for plants in the Australian Wet Tropics.

	All variable model	Single variable model
Variable	% deviance lost	% deviance explained
Annual precipitation	3.1**	23.8**
Maximum temp. warmest period	0.5*	3.4**
Annual mean radiation	0.6*	16.6**
C3 megaphyll growth index	0.5*	16.7**
C3 mesophyll growth index	0.7**	11.9**
Soil PAW holding capacity	2.0**	10.1**
Soil nutrient status	0.3*	4.8**
Geographic distance	0.3**	7.7**

*P<0.01;

**P<0.001; PAW = Plant available water; temp. = temperature.

For the full model of plant community compositional dissimilarity, the number of site pairs = 138,601, the intercept = 0.349, the null deviance = 48,010, the residual deviance = 31,568, the deviance explained = 34.2% and the model *P*-value <0.001.

We combined the model predictions of species richness for all 1,397,833 communities (grid cells) across the region with the model predictions of compositional dissimilarity between each pair of communities, to predict the current composition of every community using the *Dynamic*FOAM algorithm [26]. Across the ten replicate *Dynamic*FOAM solutions generated for the region, the proportion of species occurrences correctly predicted was 0.406 (±5.2×10^−3^) with correctly predicted occurrences significantly (*P*<0.01) more frequent than random, while the mean absolute error in predicted Sorensen’s dissimilarity between site pairs was 0.050 (±1.9×10^−4^) (Fig. S3).

**Figure 5:**
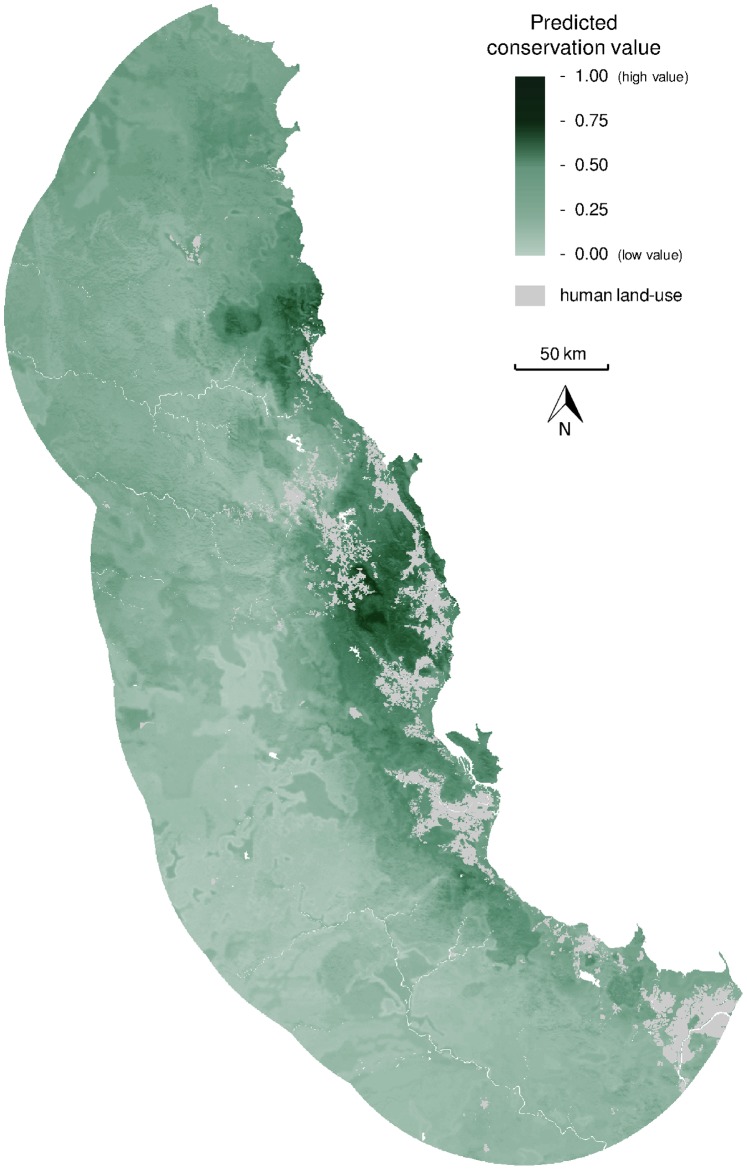
Predicted conservation value. Predictions of the overall conservation value for plant diversity of each community (grid cell) in the Australian Wet Tropics, as synthesised across the four different elements of diversity we considered (from Fig. 3).

Applying the predictions of the composition of every community (i.e. grid cell) across the region, we calculated three biodiversity metrics. First, we found that communities predicted to contain the rarest species (lowest median area of occurrence) were located in high altitude locations within the tropical forests (Fig. 3B). However, communities with the greatest predicted density of species endemic to the surrounding area of 30 km radius were concentrated in two locations: (1) the North Johnstone River in the Atherton Tablelands area, and (2) the Daintree rainforest around Thornton Peak (Fig. 3C). Finally, communities containing the largest number of “primitive” angiosperm families (described in [6], [39]) were predicted to occur more evenly across the area of wet tropical forest (Fig. 3D). When the three attributes of plant biodiversity considered here (Fig. 3) were combined with species richness (Fig. 3A) in a single index of conservation value, habitat within two areas (Atherton Tablelands, Daintree rainforest) were predicted to have the greatest overall value for conserving plant diversity in the Australian Wet Tropics (Fig. 5).

## Discussion

### Drivers of Diversity

To identify conservation and management actions that are likely to best retain the diversity of tropical forests over time, we need to rapidly improve our understanding of current biodiversity in these systems. Here we have demonstrated how macroecological modelling can overcome shortfalls in our knowledge of diversity in tropical forests [40], providing valuable new insight into current patterns plus identifying priority areas for conservation and management. Although the Australian Wet Tropics are some of the world’s best studied tropical rain forest, the same macroecological modelling approaches can be successfully applied in systems where biological information is much more limited, as the proportion of occurrences correctly predicted by the method is relatively insensitive to the amount of community survey data included in the analyses, at all but extremely low levels of input data [26].

For the Australian Wet Tropics, our continuous predictions of species richness, compositional dissimilarity and community composition for the 4,313 vascular plant species recorded in the region highlight which areas are likely to contain the greatest species richness (Fig. 3A), the most rare species (Fig. 3B), the greatest concentration of endemic species (Fig. 3C), and the greatest representation of “primitive” angiosperm families (Fig. 3D).These continuous predictions of plant diversity offer substantial advances over previous categorical assessment of vegetation patterns in the Australian Wet Tropics [12], [13].

For both the models of species richness and compositional dissimilarity, annual precipitation was the most important individual predictor (Tables 1,2; Figs. 2B, 4A), supporting substantial previous research in both the Australian Wet Tropics and other tropical forests [4], [15], [41]. Outcomes for these tropical forests under climate change are therefore likely to strongly depend upon the nature of any concomitant changes in precipitation [16], [42]. Other important explanatory variables were also shared by the macroecological models of species richness and compositional dissimilarity, including annual mean radiation and C3 megaphyll plant growth index (Tables 1,2; Fig. 2,4). Although both of these variables incorporate components of energy and temperature [30], they are also likely to be important through their relationship with moisture availability.

### Spatial Projection of Plant Diversity

Here we have demonstrated how the predictions of the species richness and compositional dissimilarity models can be combined using *Dynamic*FOAM [26], to predict the composition of every community across the region (n = 1,397,833). These complete metacommunity predictions can then be analysed in numerous ways to identify both major patterns in diversity and priorities for conservation or management. For example, in the Australian Wet Tropics, the communities predicted to contain the most rare species are not consistently those with the greatest species richness, but rather are areas with combinations of both moderately high species richness and high compositional dissimilarity to all other communities (Figs. 3A,B).

Another useful application of the predictions of community composition is in identifying areas of high species endemism. Areas that possess large numbers of endemic species are of particularly high value for conservation, through minimising the threat of extinction for the endemic species occurring there and because these areas may be important refuges for many other species under climate change [43], [44]. Our analyses of species endemism within surrounding areas of 30 km radius identified two major centres of endemism for vascular plants: the North Johnstone River in the Atherton Tablelands, and; the Daintree rainforest around Thornton Peak (Fig. 3C). Interestingly, these predictions generally align with those of Nix & Switzer [14], who identified two large rainforest refugia in the same areas of the Australian Wet Tropics, by modelling the distribution of “rainforest”, as a vegetation category, back to the last glacial maximum.

The Australian Wet Tropics flora has particular value globally for the large representation of “primitive” angiosperm families (13 out of 28) as described by Metcalfe and Ford [6], [39]. Here we combined our predictions of plant community composition across the region with the identities of the 13 primitive angiosperm families, to quantify the predicted number of primitive angiosperm families occurring within each community (grid cell) (Fig. 3D). While the density of primitive angiosperm families corresponds roughly with overall plant species richness (Fig. 3A), communities in which primitive angiosperm families are best represented are not consistently those with the most species.

### Conservation and Management Implications

Identifying priority areas for conservation and management often requires consideration of a range of biodiversity features [45]. These multiple features of biodiversity may be weighted in different ways for specific conservation applications, with a range of alternative algorithms and software available to achieve this [45], [46]. Here we demonstrate how the predictions for multiple attributes of diversity emerging from our macroecological analyses (Fig. 3) can be simply synthesised into a single index of conservation value (Fig. 5). For more specific applications, other attributes of diversity could be additionally considered, the importance of specific diversity attributes could be weighted, or our predictions of diversity could be used in iterative assessment of alternative conservation/management options to incorporate complementarity between areas. Here, however, we simply demonstrate the potential for deriving a single measure of conservation value that is clearly composed of different elements of biodiversity that are perceived to be important.

For the Australian Wet Tropics, the Atherton Tablelands and Daintree rainforest areas were predicted to have the greatest overall conservation value for plant diversity (Fig. 5), possessing combinations of high species richness, many rare and endemic species, plus many primitive angiosperm families. The prediction of high conservation value for the Atherton Tablelands is of particular relevance for conservation and management, given the high levels of habitat loss already incurred in this area (Fig. 1). Indices of overall conservation value (Fig. 5) could be used to identify priorities for new protected areas in the region, capturing any high-value tropical forest currently outside the reserve estate. Our predicted conservation value could also be applied in targeting priority locations for management actions such as alien invasive species control measures or habitat restoration efforts, so that they best contribute to the maintenance of native plant diversity.

### Future Directions

Here we have extended our knowledge of current patterns in plant diversity for the Australian Wet Tropics, through continuous modelling and prediction of species richness, compositional dissimilarity and complete metacommunity composition. We have applied these predictions to identify current priority areas for conservation and management, however, a thorough assessment of alternative management scenarios to retain biodiversity over time requires a more dynamic modelling approach. Indeed, the models and predictions of community composition generated here could form the basis for semi-mechanistic macroecological modelling to assess spatiotemporally explicit outcomes for biodiversity under specified climate and management scenarios [47]. Such semi-mechanistic modelling can incorporate dynamic dispersal processes and could be applied to help identify adaptive management actions under climate change [48]. Improved projections for plant diversity under alternative climate and management scenarios could also contribute to improving our understanding of likely outcomes for the diverse fauna that inhabit these tropical forests [49], [50].

## Supporting Information

Figure S1 The proportion of QLD Herbarium Corveg survey sites that contain different proportions of taxa not identified to species level, and the spatial distribution of the retained and omitted data across the Australian Wet Tropics.
(DOCX)

Figure S2 The proportion of records omitted from the retained QLD Herbarium survey sites as a function of the total number of taxa recorded, and the spatial distribution of sites.
(DOCX)

Figure S3 The difference between pairwise dissimilarity from the *Dynamic*FOAM predicted community compositions for the Australian Wet Tropics and both the pairwise dissimilarity predicted by the Generalised Dissimilarity Model and the observed dissimilarity from the community survey sites.
(DOCX)
