# Supplementary material for: Identifying Priority Areas for Conservation and Management in Diverse Tropical Forests
Source: PLoS One. 2014 Feb 14;9(2):e89084. doi: 10.1371/journal.pone.0089084 (PMC3925232; doi:10.1371/journal.pone.0089084)
Supplement: Figure S1 — The proportion of QLD Herbarium Corveg survey sites that contain different proportions of taxa not identified to species level, and the spatial distribution of the retained and omitted data across the Australian Wet Tropics. (DOCX) [file pone.0089084.s001.docx]

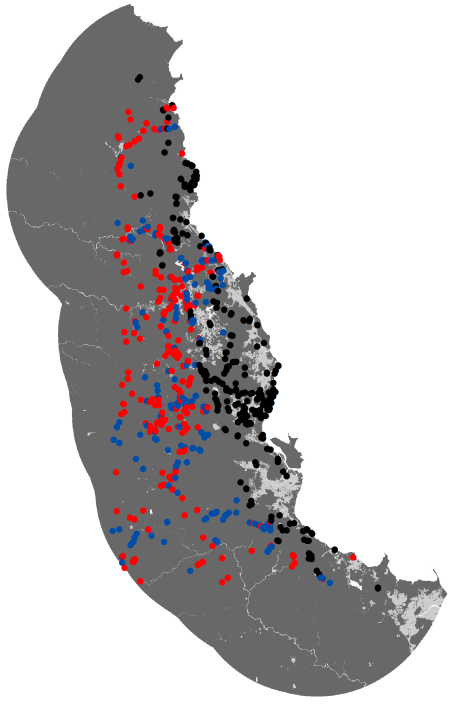


**A**

**B**

**Figure S1.** (**A**) The proportion of QLD Herbarium Corveg survey sites that contain different proportions of taxa not identified to species level, and; (**B**) the spatial distribution of the retained (blue) and omitted (red) data, as well as the CSIRO data (black), across the Australian Wet Tropics. Data were retained when the proportion of taxa unidentified to species level was < 0.1, as indicted by the red line in (A).
