# Supplementary material for: Identifying Priority Areas for Conservation and Management in Diverse Tropical Forests
Source: PLoS One. 2014 Feb 14;9(2):e89084. doi: 10.1371/journal.pone.0089084 (PMC3925232; doi:10.1371/journal.pone.0089084)
Supplement: Figure S2 — The proportion of records omitted from the retained QLD Herbarium survey sites as a function of the total number of taxa recorded, and the spatial distribution of sites. (DOCX) [file pone.0089084.s002.docx]

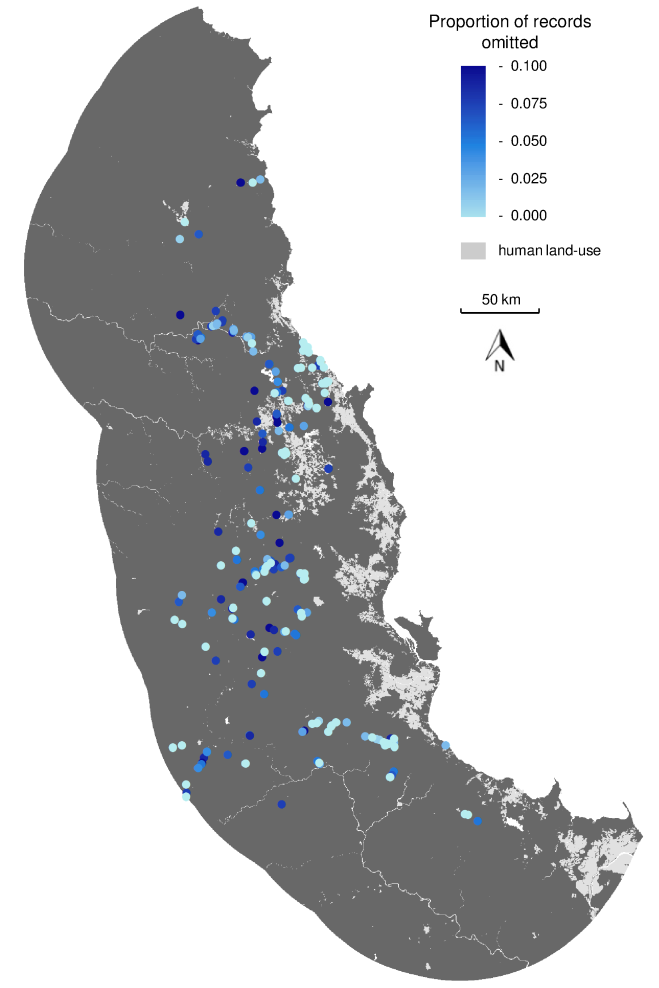


**B**


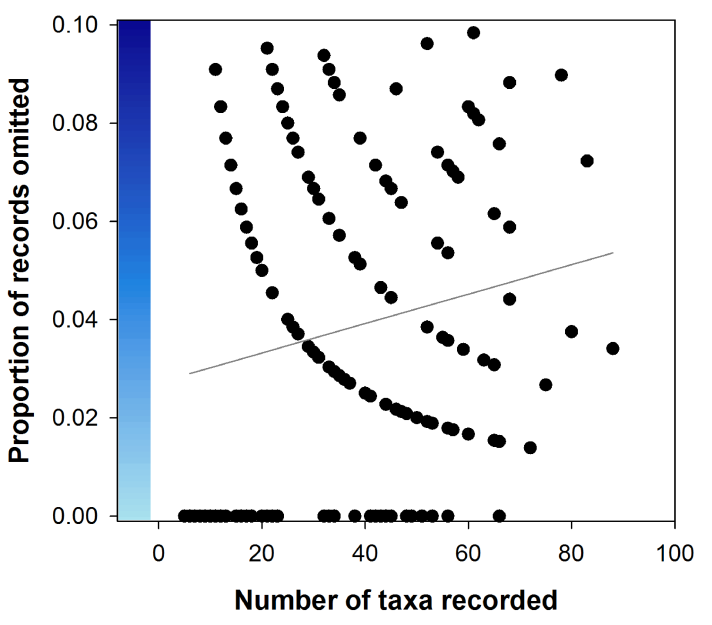


**A**

**Figure S2.** (**A**) The proportion of records omitted from those QLD Herbarium Corveg survey sites retained in the analyses as a function of the number of taxa recorded, and; (**B**) the spatial distribution of the retained Corveg sites, across the Australian Wet Tropics, with the proportion of records omitted at each site shown with the colour scale. In (A), there is a marginally significant trend for the proportion of records omitted to increase with the number of taxa recorded, as shown by the grey line (*P* = 0.011; R^2^ = 0.02).
