# Supplementary material for: Identifying Priority Areas for Conservation and Management in Diverse Tropical Forests
Source: PLoS One. 2014 Feb 14;9(2):e89084. doi: 10.1371/journal.pone.0089084 (PMC3925232; doi:10.1371/journal.pone.0089084)
Supplement: Figure S3 — The difference between pairwise dissimilarity from the DynamicFOAM predicted community compositions for the Australian Wet Tropics and both the pairwise dissimilarity predicted by the Generalised Dissimilarity Model and the observed dissimilarity from the community survey sites. (DOCX) [file pone.0089084.s003.docx]

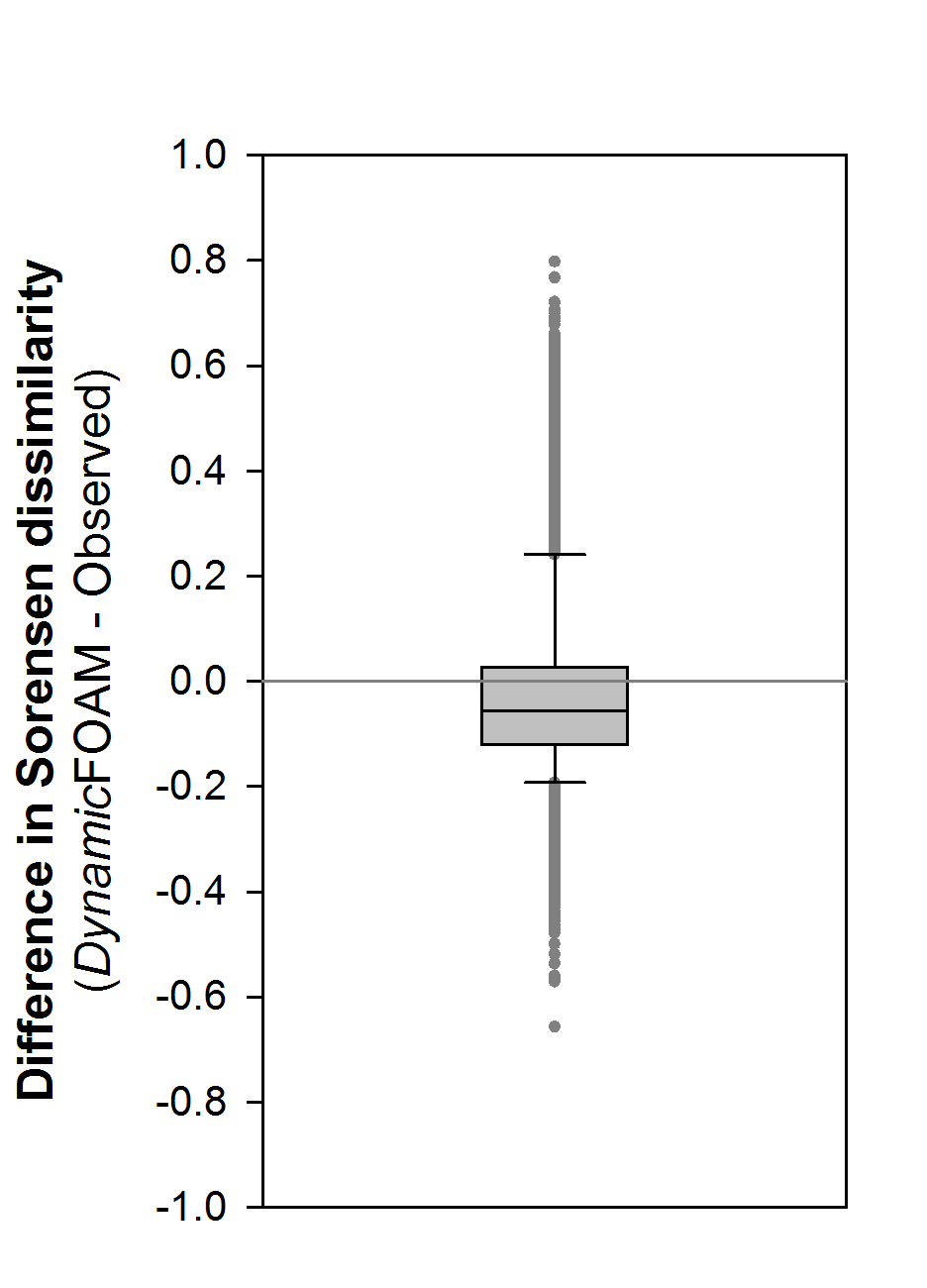

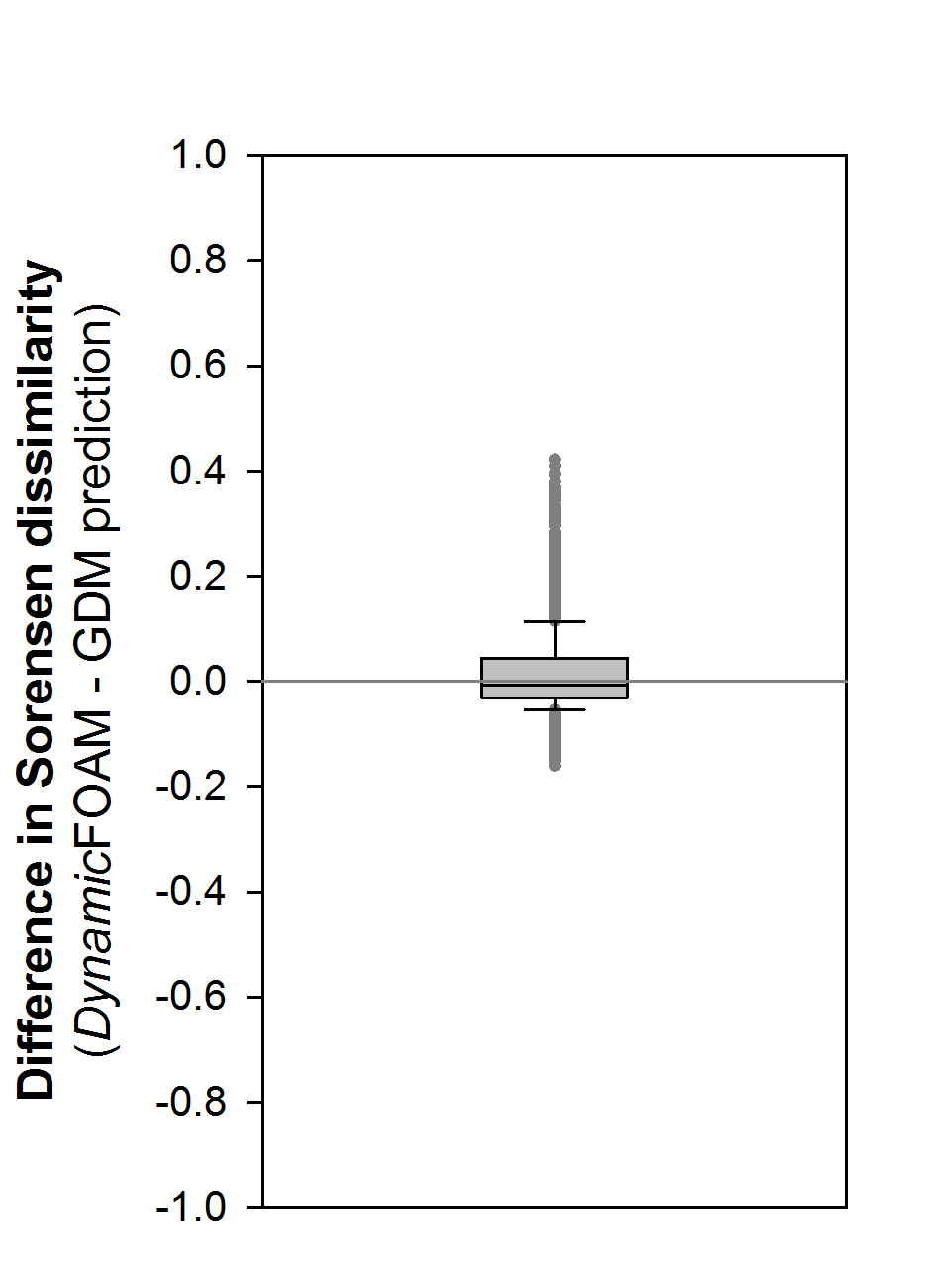


over-prediction

**B**

**A**

over-prediction

under-prediction

under-prediction

**Figure S3.** The difference between pairwise dissimilarity from the *Dynamic*FOAM predicted community compositions for the Australian Wet Tropics and: (**A**) pairwise dissimilarity predicted by the Generalised Dissimilarity Model, or ; (**B**) observed pairwise dissimilarity from the community survey sites. Positive values indicate over-prediction of compositional dissimilarity by *Dynamic*FOAM, while negative values indicate under-prediction. In (A), data are a random sample of 10,000 site pairs from a single *Dynamic*FOAM solution, while in (B), data are a random sample of 10,000 pairs of the community survey sites. Note that the objective of the *Dynamic*FOAM algorithm is to minimise the difference to the GDM predicted dissimilarities, not the original survey site dissimilarities.
